# The Ultrastructure of the Apical Organ of the Goette's Larvae of the Polyclad Flatworm *Stylochus pilidium* Indicates Homology Between Polyclad Larvae

**DOI:** 10.1002/cbin.70034

**Published:** 2025-05-15

**Authors:** Davina Düngler, Clemens Gotsis, Isabel L. Dittmann, Stefan Redl, Michael W. Hess, Bernhard Egger

**Affiliations:** ^1^ Department of Zoology University of Innsbruck Innsbruck Austria; ^2^ Institute of Neuroanatomy Medical University of Innsbruck Innsbruck Austria; ^3^ Institute of Histology and Embryology Medical University of Innsbruck Innsbruck Austria

**Keywords:** apical organ, ciliary tuft, Goette's larva, lobes, Polycladida, ultrastructure

## Abstract

Polyclad flatworms exhibit both direct and indirect development, with various larval types observed, including Müller's larva, Kato's larva, Goette's larva and Curini‐Galletti's larva. The different larval types are distinguished by shape, number of eyes and number of lobes. The Goette's larva of *Stylochus pilidium* exhibits a distinct six‐lobed morphology, with one cerebral and one epidermal eye. The posterior half of the larva features a posterior tuft and four lobes, two of which are ventrolateral and two of which are lateral. Anteriorly, a larger lobe called the oral hood is situated ventrally, opposite a smaller dorsal lobe. The larval types share a distinct feature, known as the apical organ, which is located at the anterior tip of the larvae. Here, we investigated the ultrastructure of the apical organ in Goette's larvae of the polyclad *S. pilidium*. Six apical tuft sensory (ATS) cells are at the centre of the apical organ, encircled by a ring of apical tuft gland (ATG) cell type 1 necks. These cell necks merge into two distinct gland cells that extend dorsoposteriorly and terminate posterior to the brain. Two epidermal apical tuft anchor (ATAn) cells encircle the ATS cells and the central gland cell necks. Additionally, four ATG cell type 2 necks, which are distributed symmetrically around the ATAn cells, merge into a single cell and extend ventrally to the level of the cerebral eye. The third type of ATG cells is in a circular pattern around the anchor cells, with necks in the epidermal layer. The ultrastructural arrangements of the apical organ in Goette's larva of *S. pilidium* are very similar to those of previously studied polyclad larvae, supporting the hypothesis of a common origin of larvae within Polycladida.

**Abbreviations**
28S: nuclear large ribosomal subunit
5‐HT: 5‐hydroxytryptamine
a: anterior
AO: apical organ
AT: apical tuft
ATAn: apical tuft anchor cell
ATC: apical tuft complex
ATG: apical tuft gland
ATG1: apical tuft gland type 1
ATG2: apical tuft gland type 2
ATG3: apical tuft gland type 3
ATS: apical tuft sensory cell
BM: basal membrane
BSA‐T: 1% bovine serum albumin in PBS‐T
CB: ciliary bands
CEy: cerebral eye
Ci: cilia
COI: cytochrome oxidase I
d: dorsal
DAPI: 4′,6‐diamidino‐2‐phenylindole
DATC: dorso‐apical tuft complex
DATG: dorso‐apical tuft gland
DL: dorsal lobe
DLL: dorsolateral lobe
EEy: epidermal eye
Ep: epidermis
FEy: frontal eyes
G: gut
l: left
LL: lateral lobe
M: mouth
MEy: marginal eyes
N: nucleus
Ne: neurons
Np: neuropil
OH: oral hood
p: posterior
PBS: phosphate‐buffered saline
PBS‐T: 0.1% Triton X‐100 in PBS
Ph: pharynx
PT: posterior tuft
r: right
rbd: rhabdites
SEM: scanning electron microscopy
SN: sensory neuron
TEM: transmission electron microscopy
TEy: tentacular eyes
v: ventral
Vac: vacuole
VD: vas deferens
VLL: ventrolateral lobe

## 1 Introduction

Flatworms (Platyhelminthes) are a diverse group within the spiralian taxon. Among these, the Polycladida are a subgroup of free‐living marine flatworms. Polycladida have traditionally been classified based on the presence or absence of a true ventral sucker (Cotylea with true sucker; Acotylea without true sucker) (Faubel 1983, 1984). However, recent molecular studies have revealed a more complex phylogeny among polyclads (Bahia et al. 2017; Dittmann et al. 2019a; Goodheart et al. 2023). The terms ‘Acotylea’ and ‘Cotylea’ are still used with amendments (Dittmann et al. 2019a; Goodheart et al. 2023).

Polyclads exhibit various developmental modes. While many develop directly, some species have a planktonic larval stage (Martín‐Durán and Egger 2012; Rawlinson 2014). These larvae play a crucial role in dispersal and habitat selection (Rawlinson 2014). Direct developers, on the other hand, have a similar appearance to adults but are smaller in size and sexually immature (Goodheart et al. 2023; Martín‐Durán and Egger 2012; Rawlinson 2014). Polyclad larvae come in different forms, known as Curini‐Galletti's, Goette's, Kato's and Müller's larvae (Dittmann et al. 2024; Martín‐Durán and Egger 2012; Rawlinson 2014). Traditionally, these larvae are distinguished by variations in eye number, lobe number and body shape (Martín‐Durán and Egger 2012; Rawlinson 2014). Curini‐Galletti's larvae show three lobes and one eye (Dittmann et al. 2024). Goette's larvae possess 4 lobes and 2–3 eyes (Goette 1882; Martín‐Durán and Egger 2012; Rawlinson 2014). Kato's larvae have 8 lobes and 12 eyes (Nielsen 2012). The most common polyclad larvae are Müller's larvae, which typically have 6–8 lobes and 3 eyes, although some variations are known (Martín‐Durán and Egger 2012; Rawlinson 2014).

The observed similarities among polyclad larval forms raise a fundamental question regarding their evolutionary relationships. Two main hypotheses can explain these similarities: the larvae may have descended from a common ancestor that possessed a similar larval stage. In this scenario, the observed similarities could be considered homologous traits, indicating a shared evolutionary origin. Alternatively, the similarities could be the result of convergent evolution. Natural selection may have independently favoured the development of similar larval forms in different lineages, despite their lack of a shared ancestor (Dittmann et al. 2023; Goodheart et al. 2023). To address this fundamental question of homology or convergence in polyclad larval types, detailed morphological and ultrastructural comparisons are essential. While comprehensive data already exist for the other three larval types, there are still knowledge gaps regarding acotylean Goette's larvae.

Goette's larvae are relatively small, measuring around 100 µm in length. They exhibit an hourglass‐like shape, with a distinct anterior region and a broader posterior section. These larvae possess 1–2 eyes, located at the anterior end. Additionally, they have four lobes: a dorsal hood, two lateral lobes (LLs) and a dorsal lobe (DL) (Goette 1882; Ruppert 1978). The entire body surface of Goette's larvae is covered with cilia. These cilia are particularly long on the lobes, forming distinct ciliary bands. Goette's larvae possess a mouth opening, a pharynx and a gut (Lacalli 1982, 1983). At the anterior end, a brain is present. A posterior tuft, a cluster of cilia, is found at the posterior end. Another defining characteristic shared by these larval stages is the presence of an apical organ, a specialised structure located at the anterior tip of the larvae (Dittmann et al. 2023, 2024; Lacalli 1982, 1983; Ruppert 1978).

For Goette's larvae, knowledge about the exact cellular arrangement of the apical organ is scarce, but previous studies on polyclad larvae have shown a common pattern of ultrastructural organisation of the apical organ, consisting of at least three distinct cell types: ciliated apical tuft sensory (ATS) cells, apical tuft gland (ATG) cells and apical tuft anchor (ATAn) cells. The centrally located sensory cells could be observed to be surrounded by glandular cells, which are themselves embedded in anchor cells (Dittmann et al. 2023; Lacalli 1982, 1983; Ruppert 1978). This suggests a potentially conserved cellular arrangement within the apical organ across polyclad larvae, highlighting the importance of further investigation in Goette's larva to understand potential variations or homologies.

Larval stages are not unique to polyclads; similar larval forms can be found in other spiralian animals such as annelids, molluscs or entoprocts (Nielsen 2012). These larval forms, known as trochophore larvae, share some common characteristics with polyclad larvae, including the presence of an apical tuft (AT) and ciliary bands, a free‐living lifestyle and a spiral cleavage pattern (Dittmann et al. 2023; Nielsen 2012). This raises the question of how these larval types are related. Are they homologous, meaning they share a common ancestor and therefore a similar larval form? Or did they evolve independently (convergently) due to similar environmental pressures, resulting in a resemblance? Is the apical organ in polyclad larvae homologous (shared ancestry) or convergent (independent evolution) to that found in trochophore larvae? Answering this question could clarify whether the most recent common ancestor of Spiralia possessed a larval form or if the biphasic life cycle evolved independently multiple times.

This study aims to address the knowledge gap regarding the homologous or convergent nature of polyclad larval forms by investigating the larval morphology and ultrastructure of the apical organ in Goette's larva of the acotylean polyclad *Stylochus pilidium*. By comparing the newly acquired data of the apical organ of Goette's larvae with existing knowledge of other polyclad larval stages, we aim to achieve a broader understanding of the apical organ's development, evolutionary history and its role in the evolution of polyclad larvae themselves.

## 2 Materials and Methods

### 2.1 Animals

A single adult *S. pilidium* was collected under a stone lifted from the sand of a rubble mound breakwater near Valencia, Spain (39.51081° N, −0.31864° E) in August 2022. The animal was kept in artificial seawater in a laboratory in Innsbruck, Austria, at 18°C without feeding, where it laid egg plates in the culture container. Larvae were collected about 1 week later from the water column on the day of hatching or up to 3 days later.

### 2.2 Light Microscopy

The adult animal was photographed on an object slide with a small amount of seawater using a Leica MZ16F microscope with a Leica DFC450 C camera attached. Some of the hatched larvae were visualised in a squeeze preparation using a Leica DM5000 B microscope with a Leica DFC490 camera attached.

For fluorescent stainings, 1–3 days old larvae were anaesthetised for 10 min in a 7.14% magnesium chloride hexahydrate solution, fixed for 1 h at room temperature in 4% formaldehyde in 0.1 M phosphate‐buffered saline (PBS), rinsed three times in PBS and then soaked for 45 min in BSA‐T (1% bovine serum albumin and 0.1% Triton X‐100 in PBS). Larvae were incubated overnight at 4°C in a 1:3000 solution of a primary rabbit anti‐5‐HT antibody (Sigma) in BSA‐T. After rinsing five times in PBS‐T at room temperature and soaking for 2 h in BSA‐T, larvae were incubated for 1 h in BSA‐T containing 1:250 goat anti‐rabbit Alexa Fluor 488 secondary antibody (Thermo Fisher Scientific), 1:250 Atto‐565‐conjugated phalloidin (Sigma) and 1:10000 DAPI (Thermo Fisher Scientific). After rinsing several times in PBS‐T and overnight at 4°C, animals were coverslipped in Vectashield (Vector Labs). Confocal stacks were made on a Leica TCS SP5 II microscope. Stacks were handled with Fiji (Schindelin et al. 2012) and confocal figures were made with GIMP (https://gimp.org).

### 2.3 Molecular Work

After egg laying, the adult animal was stored in 100% ethanol at −20°C for later DNA extraction. DNA was extracted using a phenol‐chloroform protocol (see Dittmann et al. 2019b for details). The partial large ribosomal subunit (28S) was amplified with the primers 28S_1F and 28S_6R (Larsson and Jondelius 2008) using the following PCR conditions: 94°C 5 min; 35 cycles of 94°C 30 s, 53°C 30 s, 72°C 2 min; 72°C 10 min; store at 16°C. Sequencing of successful amplicons was done by Microsynth (Austria).

### 2.4 Scanning Electron Microscopy (SEM)

Larvae were relaxed in 7.14% magnesium chloride hexahydrate, fixed after the protocol of Eisenman and Alfert (1982), followed by dehydration with acetone and critical point drying with a BAL‐TEC CPD 030. To prevent drying of artefacts, the specimens were pipetted onto polystyrene pots or filter paper sachets placed in ethanol. These containers were then closed and placed into the pressure chamber and flooded with liquid CO_2_. After successful dehydration, the specimens were mounted on 0.5″ aluminium stubs (Agar Scientific Ltd) using sticky conductive standard carbon tabs (Science Services). An electromagnetically charged preparation needle was used to carefully orient the larvae on the carbon tabs, minimising surface damage. The preparation needle was charged with a piece of polyethylene material. Finally, the samples were sputter‐coated with gold using a BAL‐TEC MED 020 Coating system (layer thickness: 20 nm; process current: 30.0 mA; process pressure: 5.0e^−2^ mbar). For examination and visualisation, a Zeiss DSM 982 Gemini Scanning Electron Microscope was used. Image processing was done with Adobe Photoshop 7.

### 2.5 Transmission Electron Microscopy (TEM)

One‐to‐three days old larvae were relaxed in 7.14% magnesium chloride hexahydrate, fixed for electron microscopy after Eisenman and Alfert (1982) (see Dittmann et al. 2024) and dehydrated in acetone. Dehydrated larvae were embedded in EPON (see Salvenmoser et al. 2010; Gammoudi et al. 2016). Two specimens were analysed, one using sagittal sections (Specimen #1) and the other using cross‐sections (Specimen #2). Sections were cut on a Leica ultracut UCT microtome (semithin sections: 0.35 µm and ultrathin sections: 80 nm). Semithin sections were stained with methylene blue azure II disodium tetraborate decahydrate (Richardson et al. 1960), and ultrathin sections were contrasted with lead citrate. Sections were examined with a Zeiss Libra 120 energy filter transmission electron microscope operated at 80 kV. ImageSP (Tröndle) and Adobe Photoshop 7 were used for image processing, and the illustrations were made with Adobe Illustrator.

## 3 Results

### 3.1 Species Determination

**Figure 1:**
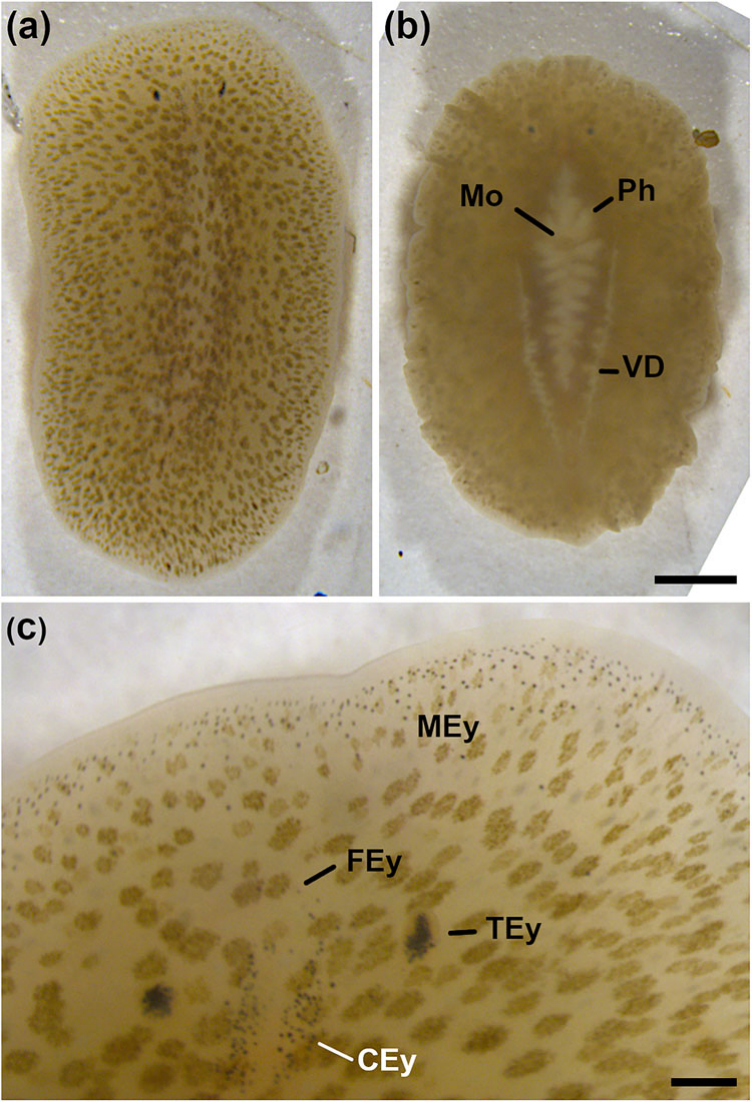
Live images of an adult *Stylochus pilidium*. (a) Whole animal, dorsal surface. (b) Whole animal, ventral surface. (c) Anterior part, dorsal surface. CEy, cerebral eyes; FEy, frontal eyes; M, mouth; MEy, marginal eyes; Ph, pharynx; TEy, tentacular eyes on tentacle; VD, vas deferens. In all panels anterior to the top. Scale bars: 1 mm (a–b), 200 µm (c).

The live specimen was ca. 7 mm in length, depending on the animal's state of elongation. The measurements were between ca. 6 mm in a resting state (Figure 1b) and 7.6 mm in full movement, and its width was between 3.6 mm and 3.8 mm. The body colour was a transparent beige‐brown (Figure 1a–c) dotted with round and oval brown spots (Figure 1a), where each spot consisted of several smaller dots (Figure 1c). Two pointed tentacles were situated besides the brain, with each tentacle bearing many eyes (Figure 1c). On the brain, two broad rows of cerebral eyes were found (Figure 1c). At the edge of the animal, marginal eyes circled it in broad rows at the anterior third (Figure 1c), and in thinner rows more posteriorly. Between cerebral eyes and anterior marginal eyes, a few frontal eyes could be seen (Figure 1c). On the ventral side, the pharynx occupied the middle half of the animal and had about nine diverticula, with the mouth opening found behind the first two pharynx diverticula (Figure 1b). The vasa deferentia started at the level of the mouth and went posterior in an approximate wedge shape, ending at about one‐seventh before the posterior tip of the animal (Figure 1b).

The partial 28S sequence obtained was 1.584 bp in length and is available under the accession number PV069488 on GenBank. Blasting (blastn) the sequence revealed a 99.89% identity (913 of 914 bp identical) with a published sequence attributed to *Stylochus oculiferus* (HQ659007.1) from Florida, and a 99.26% identity (1349 of 1359 bp identical) with a published sequence from *Stylochus stellae* (MN384692.1) from Valencia, Spain. Having a closer look at the *S. stellae* sequence shows that 9 of the 10 different basepairs concern ambiguous sites (i.e., an unresolved nucleotide like N at these positions), and the remaining different basepair is an additional nucleotide in the sequence of *S. stellae* compared to all other *Stylochus* 28S sequences.

### 3.2 General Body Morphology of Larvae

**Figure 2:**
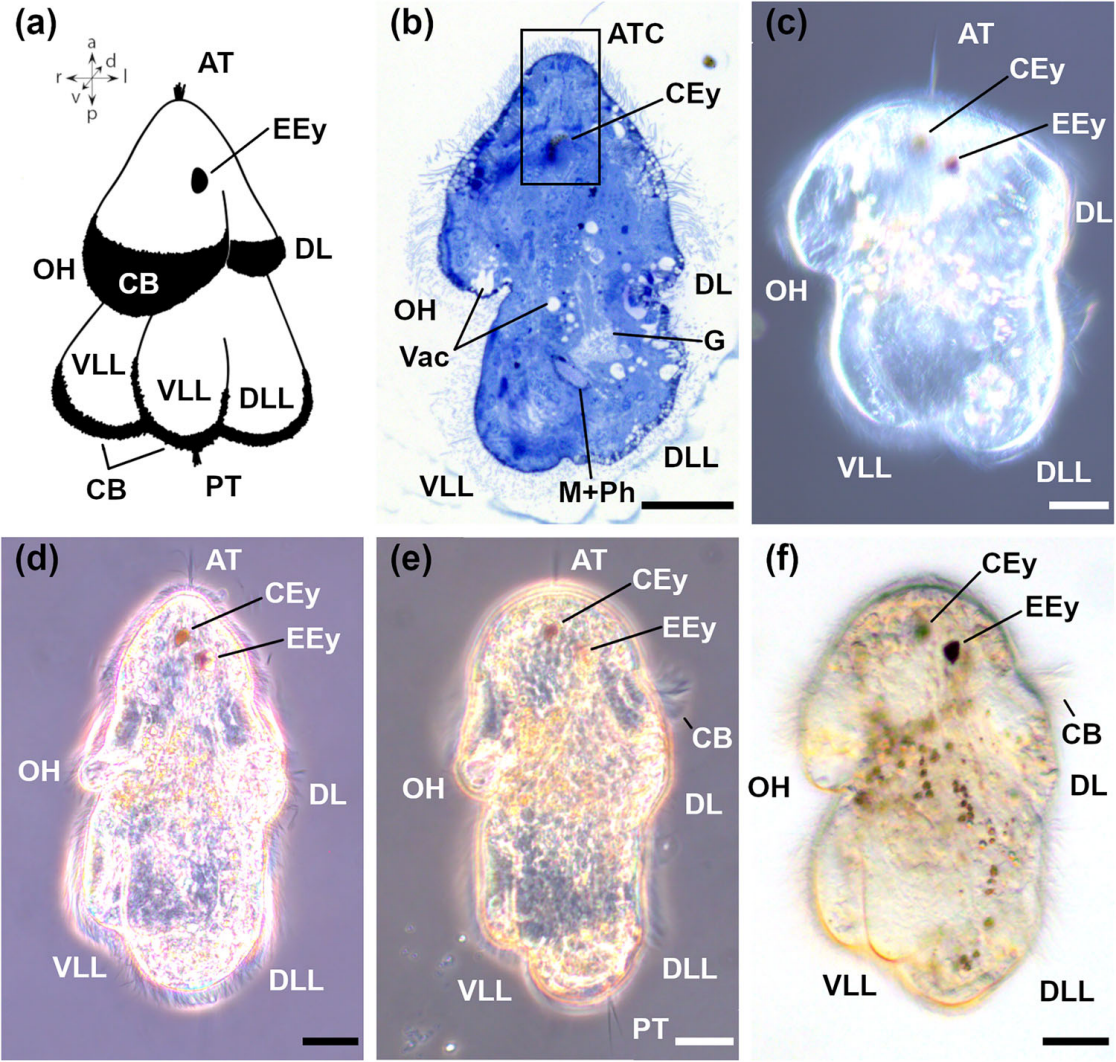
Morphological overview of 1–3 days old Goette's larvae of *Stylochus pilidium*. (a) Schematic drawing. Orientation of the drawn larva indicated by arrows (a, anterior; d, dorsal; l, left; p, posterior; r, right; v, ventral). (b–f) Lateral view, ventral is left, anterior is up. (b) Midsagittal section (Specimen #1). (c–e) Phase contrast. (f) Differential interference contrast. AT, apical tuft; ATC, apical tuft complex; CB, ciliary bands; CEy, cerebral eye; DL, dorsal lobe; DLL, dorsolateral lobe; EEy, epidermal eye; G, gut; M, mouth; OH, oral hood; Ph, pharynx; PT, posterior tuft; Vac, vacuole; VLL, ventrolateral lobe. Asterisks denote artefacts. Scale bars: 20 µm. Adapted with permission from Düngler (2024).

**Figure 3:**
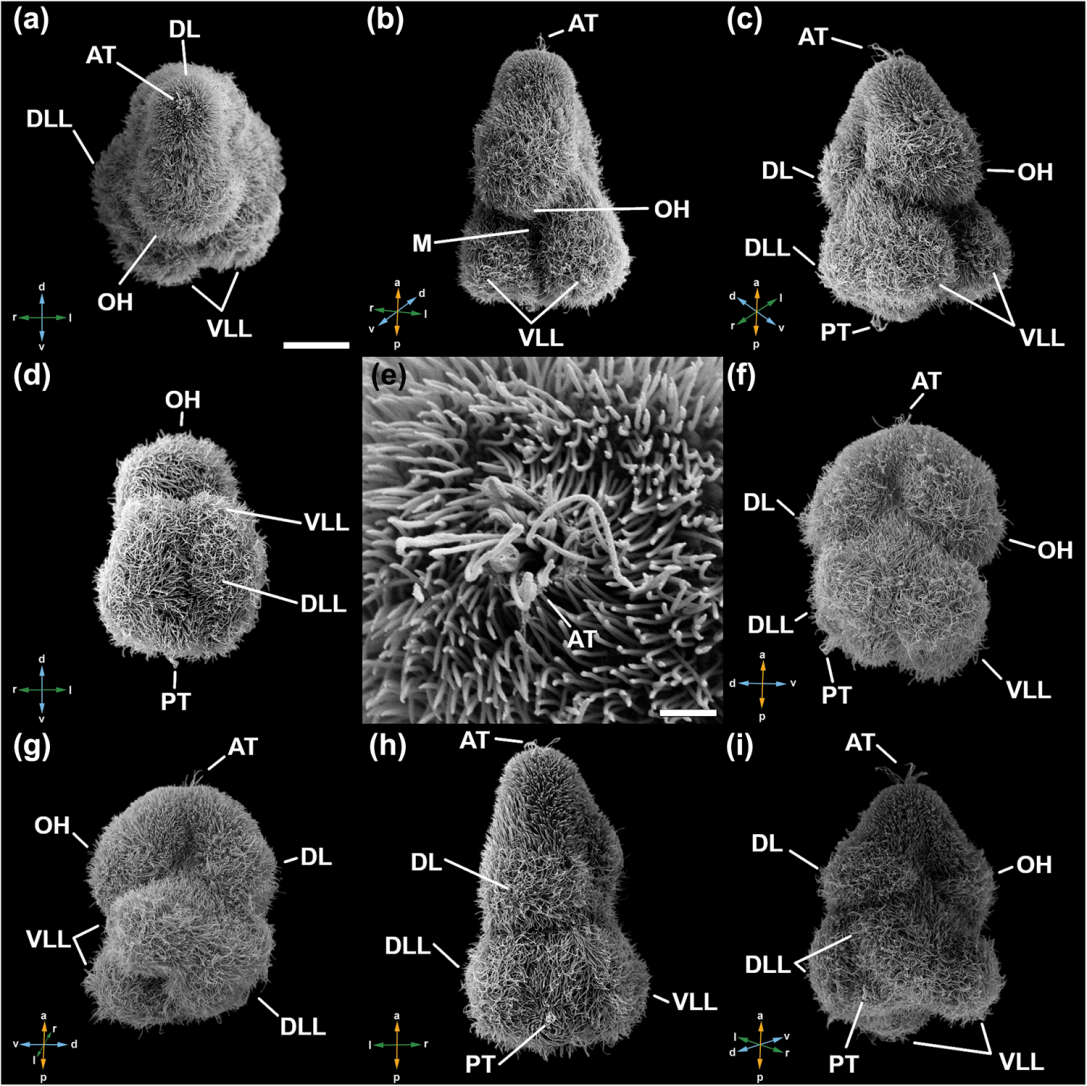
Scanning electron microscopical images of 1–3 days old Goette's larvae of *Stylochus pilidium*. (a) Apical view. (b) Ventral view. (c) Ventrolateral view. (d) Posterior view. (e) Detail of AT in (a). (f) Lateral view (right side). (g) Lateral view (left side). (h) Dorsal view. (i) Dorsolateral view. Anterior is to the top for all animals except for (a), where dorsal is to the top, and (d), where ventral is to the top. Coloured arrows show orientation: a, anterior; d, dorsal; l, left; p, posterior; r, right; v, ventral. AT, apical tuft; DL, dorsal lobe; DLL, dorsolateral lobe; M, mouth; OH, oral hood; PT, posterior tuft; VLL, ventrolateral lobe. Scale bars: 20 μm (a–d, f–i) and 2 μm in (e). Adapted with permission from Gotsis (2024).

The Goette's larva of the flatworm *S. pilidium* is shaped as an hourglass but with a narrower anterior and broader posterior end (Figures 2a‐c,f and 3c,f,g,i). The length of the larva fixed for electron microscopy is about 100 µm (± 4 µm, *n* = 9; measured from the anterior to the posterior tip); the widest region is from the tip of the oral hood (OH) to the DL (from ventral to dorsal) and measures 57 µm (± 6 µm, *n* = 11). The widest region from left to right is 54 µm (± 5 µm, *n* = 11; measured at the level of the ventrolateral or at the LLs). Live animals are about 30–40% larger than animals fixed for electron microscopy. The entire larva is covered with cilia and comprises six lobes (Figure 3a–i).

In the anterior half of the body, the larva features a larger OH ventrally and a smaller DL, with a clear separation between them (Figures 2a–c and 3a,f–g,i). Anteriorly, two eyes are present: a cerebral eye (CEy) located slightly to the left side and an epidermal eye (EEy) situated on the left side in the epidermis (Figure 2c–f). The AT is located at the anterior tip (Figures 2a,c–e and 3b–c,e–i).

The posterior half of the larva has four lobes, two ventrolateral lobes (VLLs) and two dorsolateral lobes (DLLs) (Figure 3d,g,i). At the posterior tip, between the two LLs, a posterior ciliary tuft (PT) can be found (Figures 2a,e and 3c,f). The mouth opening is enclosed on both sides by a VLL and anteriorly by the OH (Figure 2c). The pharynx emerges from the mouth opening and transitions into a muscular gut that extends towards the anteriorly positioned brain (Figure 2b).

### 3.3 Fluorescent Stainings

**Figure 4:**
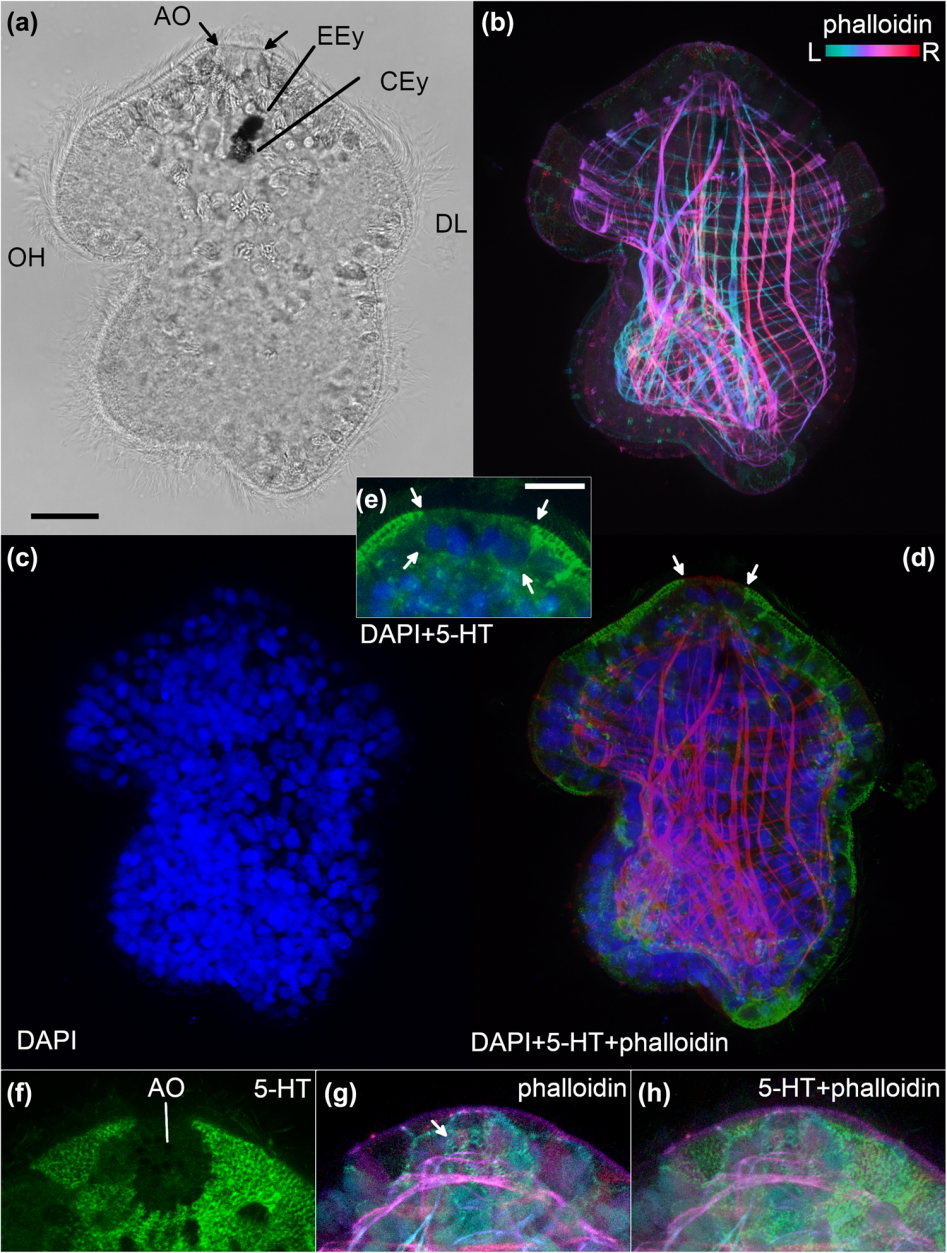
Whole‐mount confocal fluorescent stainings of 1–3 days old Goette's larvae of *Stylochus pilidium*. (a–e) depict the same individual, and (f–h) the apical region of a second individual. (a) Differential interference contrast bright‐field image. (b) Depth‐coded phalloidin staining for F‐actin, where blue is more left and red is more right. (c) DAPI staining for nuclei. (d) Merged image of F‐actin (red), DAPI (blue) and 5‐HT background resorption (green). Arrows point at the apical borders of the apical organ. (e) Detailed view of the apical region showing a merged image of DAPI (blue) and 5‐HT background resorption (green). Arrows point at the epithelial part of the apical organ. (f) Unstained circular area of the 5‐HT background resorption delineates the apical organ region. (g) Depth‐coded phalloidin staining showing the cytoskeleton rings (arrow) of the apical‐most layer. (h) Merged image of (f) and (g). AO, apical organ; CEy, cerebral eye; DL, dorsal lobe; Eey, epidermal eye; OH, oral hood. The anterior is up, and the ventral is left in all images. Scale bars: 20 µm for (a–d) and 10 µm for (e–h).

We made triple stainings of nuclei (DAPI), F‐actin musculature (phalloidin) and the serotonergic nervous system (5‐HT) on fixed whole‐mount larvae (Figure 4a) to visualise prominent body parts of the larvae. While the DAPI (Figure 4c–e) and phalloidin (Figure 4b,d,g–h) stainings worked as intended, we mainly observed background resorption of the secondary antibody in the 5‐HT stainings, which was remarkably missing in the area of the apical organ (Figure 4d–f,h) and therefore useful as a negative marker. In an approximately isometric view, the 5‐HT background staining delineated the apical borders of the apical organ (Figure 4f,h); within this ring, phalloidin staining showed a multitude of small rings (Figure 4g), which are likely indicating the cytoskeleton of gland necks and ATS cells of the apical organ.

### 3.4 Ultrastructure of the Apical Organ

**Figure 5:**
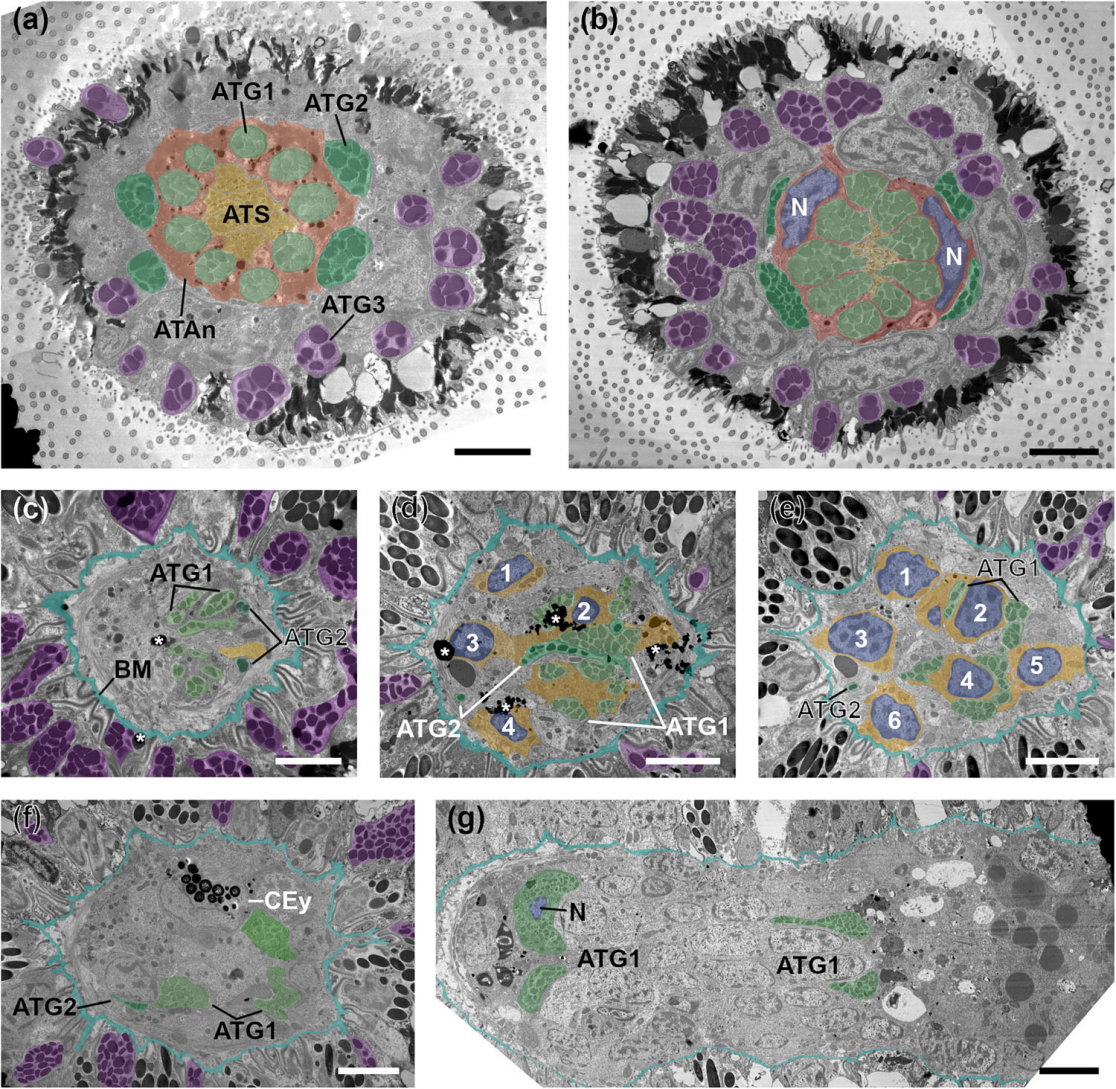
Electron micrographs of serial cross‐sections through the apical region of an 1–3 days old Goette's larva (Specimen #2) of *Stylochus pilidium*. (a) Anterior‐most section with ATS cells encompassed by two ATAn cells with cell necks of ATG1 cells, and further out ATG2 and ATG3 cell necks. (b) Slightly more posterior section with the two ATAn nuclei being visible. (c) More posterior section on the level of the BM and ATG1 cell necks on the dorsal side. (d–e) More posterior sections showing ATS cells and their numbered nuclei, as well as ATG gland cell necks. (f) Cross‐section at the level of the cerebral eye. (g) ATG1 nucleus beneath the brain. ATAn, apical tuft anchor cell (red); ATG1, apical tuft gland cell type 1 (light green); ATG2, apical tuft gland cell type 2 (dark green); ATG3, apical tuft gland cell type 3 (purple); ATS, apical tuft sensory cell (yellow); BM, basal membrane (turquoise); CEy, cerebral eye; N, nucleus (blue). Asterisks mark contrasting artefacts. Orientation: ventral is on the left side. Scale bars: 2.5 µm. Adapted with permission from Düngler (2024).

**Figure 6:**
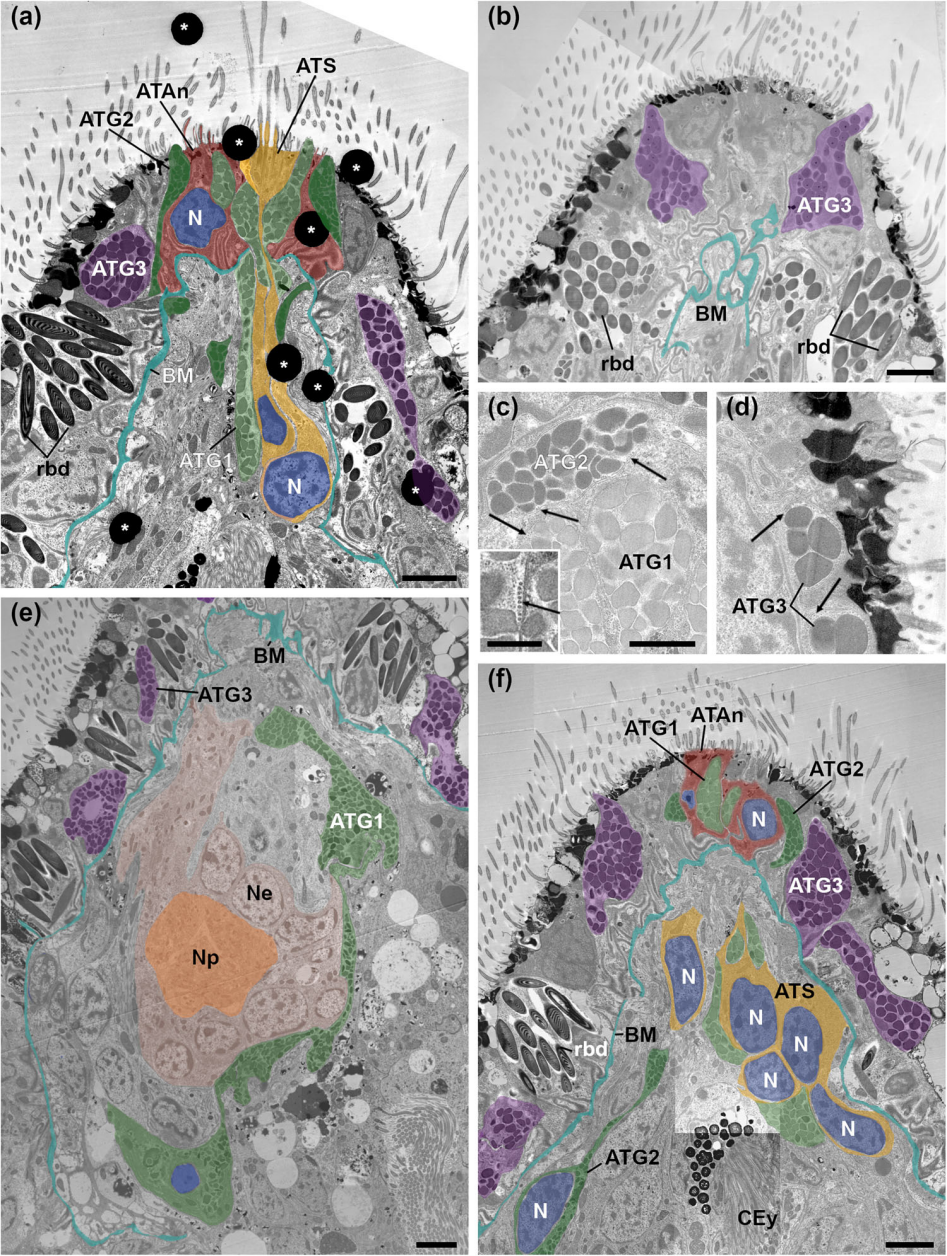
Electron micrographs of sagittal sections of the apical organ of an 1–3 days old Goette's larva (Specimen #1) of *Stylochus pilidium*. (a) Median section of the apical organ. (b) Detail of ATG3 cells at the anterior pole. (c) Details of ATG1 and ATG2 cells with granules. Inset: enlarged microtubuli. (d) Detail of ATG3 granules. (e) Detail of the ATG1 cell and its nucleus, lying posterior to the brain. (f) ATS cells with nuclei at the level of the cerebral eye. ATAn, apical tuft anchor cell (red); ATG1, apical tuft gland cell type 1 (light green); ATG2, apical tuft gland cell type 2 (dark green); ATG3, apical tuft gland cell type 3 (purple); ATS, apical tuft sensory cell (yellow); BM, basal membrane (turquoise); CEy, cerebral eye; N, nucleus (blue); Ne, neurons (light peach); Np, neuropil (peach); rbd, rhabdites. Arrows point to microtubules. Asterisks mark contrasting artefacts. Orientation: ventral is on the left side, anterior to the top. Scale bars: 2.5 µm; inset in (c): 500 nm. Adapted with permission from Düngler (2024).

**Figure 7:**
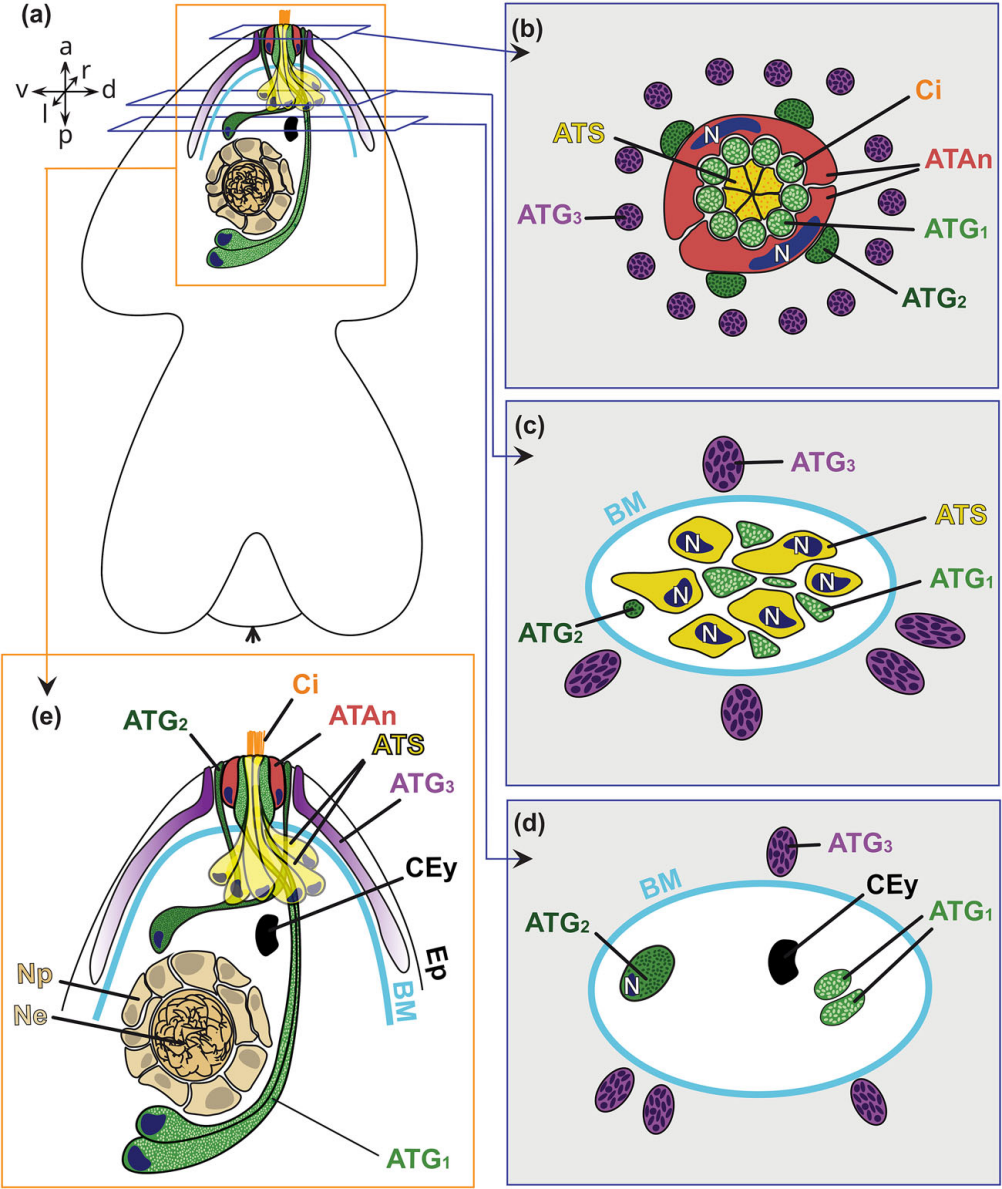
Reconstruction of the apical organ of an 1–3 days old Goette's larva of *Stylochus pilidium*. Orientation of the drawn larva indicated by arrows (a, anterior; d, dorsal; p, posterior; v, ventral). (a) Lateral view of the larva (left side). (b–d) Cellular arrangement of the indicated layers in (a). (e) Magnification of the squared area indicated in (a). ATAn, apical tuft anchor cell (red); ATG1, apical tuft gland cell type 1 (light green); ATG2, apical tuft gland cell type 2 (dark green); ATG3, apical tuft gland cell type 3 (purple); ATS, apical tuft sensory cell (yellow); BM, basal membrane (turquoise); CEy, cerebral eye; Ci, cilia (orange); Ep, epidermis; N, nucleus (blue); Ne, neurons (light peach); Np, neuropil (peach); rbd, rhabdites. Adapted with permission from Düngler (2024).

The apical organ extends from the anterior‐most part of the larva towards posterior below the brain, passing by both the brain and the cerebral eye. It measures about 30 µm from anterior to posterior and contains AT ciliated sensory (ATS) cells, ATAn cells and three types of ATG cells (Figures 5, 6, 7), which can be distinguished by size, electron density and type of granules (Table 1 and Figure 6c–d).

**Table 1:** Differences between the distinct cell types of the apical organ.

Cell type	Granules	Microtubules	Number	Electron density	Comment
ATG1	423 ± 75 nm, *n* = 29	Peripheral	2	Low	Run dorsally; furcated
ATG2	434 ± 138 nm, *n* = 34	Peripheral	1	Medium	Run ventrally, furcated
ATG3	601 ± 117 nm, *n* = 40	Peripheral	Undetermined	High	Epidermal
ATS	—	—	6	Low	Multiciliated
ATAn	—	—	2	Low	Epidermal

The apical organ in Goette's larva of *S. pilidium* shows a well‐organised and symmetrical cellular arrangement in its anterior tip (Figure 5a–b). The centre is made of a cluster of six ATS cell necks, surrounded by two ATAn cells. The presence of several ciliary rootlets within one ATS cell indicates multiciliation (Figures 6a and 7e). Nine ATG1 cell necks are embedded in the ATAn cells, while four ATG2 cell necks are positioned symmetrically around them (Figure 5a). ATAn cells are situated in the epidermal layer above the basal membrane, and their nuclei are located right above the basal membrane (Figures 6a and 7a,e). Compared to epidermal cells, the ATAn cells show less pigmentation and no vacuoles (Figures 5a and 6a). ATG3 cells reside outside this central arrangement, forming a circular pattern in the epidermal layer (Figures 5a–b and 7b). The gland necks of all three ATG cell types open to the exterior at the anterior tip (Figures 6a–b,f and 7a,e).

Approximately 10 µm from the tip, towards the basal membrane, the symmetry begins to break down (Figure 5c–g). ATG1, ATG2 and ATS cells extend through the basal membrane and elongate into different directions. The six ATS cell bodies with their nuclei are located anterior to the cerebral eye (Figures 5d–e and 7c). Nine ATG1 cell necks merge dorsal to the ATS cells into two separate cell bodies, which extend posterior and ventral to the brain, where their nuclei reside (Figure 6e). Four ATG2 cell necks merge in the epidermis into two broader cell bodies, pass the basal membrane and further merge into a single‐cell body (Figures 5a–b and 6c). This cell extends ventrally towards the level of the eye, where the ATG2 cell nucleus is located (Figures 6f and 7d). ATG3 cell necks are distributed throughout the epidermis, and the cells terminate at the level of the brain. The exact number of ATG3 cells remains unclear due to the undetermined position and number of the nuclei (≥ 1).

A common feature shared by different ATG types is the presence of peripheral microtubules (Figure 6c–d). However, these cell types differ in their electron density and granule size (Table 1). Within the ATG types, ATG1 and ATG2 cells have similar granule sizes (423 ± 75 nm, *n* = 29 for ATG1 and 434 ± 138 nm, *n* = 34 for ATG2). They can be distinguished by their electron density. Granules of ATG1 cells have the lowest electron density, while the electron density of ATG2 granules is higher. Granules of ATG3 cells have the highest electron density and possess the largest granules (601 nm, *n* = 40).

## 4 Discussion

In Müller's larva of *Prostheceraeus crozieri* and Curini‐Galletti's larva of *Theama mediterranea*, the apical organ consists of the apical tuft complex (ATC) and a dorso‐apical tuft complex (DATC) or a ventral array of sensory neurons and gland cells (Dittmann et al. 2023, 2024), while in Goette's larva of *S. pilidium*, the apical organ is identical with the ATC.

### 4.1 Species Determination

We identified the collected polyclad as *S. pilidium* based on its external habitus and morphology, mainly regarding the body colouration and the tentacles. Here, we provide the first partial 28S sequence of *S. pilidium*, as there is no published sequence of *S. pilidium* available to date. The partial 28S sequence of our specimen showed a high similarity with two published sequences of two different *Stylochus* species, *S. oculiferus* and *S. stellae*. Different to *S. stellae*, our polyclad showed frontal eyes (Figure 1c) and the pharynx occupied the centre of the animal (Figure 1b), instead of the anterior half as in *S. stellae* (Marquina et al. 2014). *S. oculiferus*, on the other hand, has conical tentacles, a very broad pharynx and pink and red spots (Hyman 1941). The sequence similarities can be explained by a close phylogenetic relationship between these three species. In other polyclads, nuclear marker genes like 28S are sometimes not sufficient to distinguish between different species, and only a mitochondrial marker like COI is able to assist morphological species determination and differentiation (Oya and Kajihara 2017; Kapeller et al. 2024). Future research, where both histological sections and DNA sequences from the same individual of similar *Stylochus* species will be obtained, can further clarify their interrelationships.

### 4.2 Comparison of Lobe Numbers Between Different Goette's Larvae Within Acotylea

The initial description of Goette's larva was provided by Goette (1882) in *S. pilidium*. Goette depicted the larva as a four‐lobed organism, whereas Lang (1884) described six lobes (Table 2). Lang (1884) observed that the freshly hatched larva resembled Goette's description with four lobes. At a later stage of larval development, two additional lobes emerged, resulting in a six‐lobed larva with an OH, DL, two VLLs and two LLs (Lang 1884).

**Table 2:** Comparison of lobe numbers of Goette's larva of different polyclad species. DL, dorsal lobe; DLL, dorsolateral lobe; LL, lateral lobe; OH, oral hood (reprinted with permission from Gotsis 2024).

*Stylochus aomori*	4 (OH, DL, 2 LLs)	Kato (1940)	
*Stylochus iijmai*	4 (OH, DL, 2 LLs)	Rho (1976)	
*Stylochus lateotentare*	4 (OH, DL, 2 LLs)	Lee et al. (2006)	
*Stylochus mcgrathi*	4 (OH, DL, 2 LLs)	Jennings and Newman (1996)	
*Stylochus mediterraneus*	6 (OH, DL, 4 LLs)	Gammoudi et al. (2012)	Probably 2 LLs and 2 DLLs
*Stylochus uniporus*	4 (OH, DL, 2 LLs)	Kato (1940)	
*Stylochus ellipticus*	4 (OH, DL, 2 LLs)	Allen et al. (2017)	No dorsolateral lobes visible
*Stylochus flevensis*	4 (OH, DL, 2 LLs)	Girard (1854)	
*Stylochus pilidium*	4 (OH, DL, 2 LLs)	Goette (1882)	Probably the lobe number depends on the developmental stage
	6 (OH, DL, 4 LLs)	Lang (1884), this study	
*Stylochus pygmaeus*	4 (OH, DL, 2 LLs)	Merory and Newman (2005)	
*Stylochus tauricus*	4‐5 (OH, DL, 2 LLs)	Murina et al. (1995)	
*Notoplana australis*	4 (OH, DL, 2 LLs)	Anderson (1977)	Subtle dorsal lobe

This study shows the presence of six lobes in up to 3 days old Goette's larva of *S. pilidium* (Figure 2). Most species of *Stylochus* exhibit four lobes: an OH, a DL and two LLs (Table 2). *Stylochus tauricus* hatches without lobes, gains two posterior lobes 2 h after hatching and develops four lobes 2–3 days after hatching, which increase to five lobes about 1 week after hatching (Murina et al. 1995). Similar to Lang (1884) and our findings for *S. pilidium*, *Stylochus mediterraneus* shows six lobes, with OH, DL, 2 LLs and two additional DLLs (Gammoudi et al. 2012) (Table 2). *Notoplana australis* exhibits four lobes, though a slight dorsal irregularity suggests the presence of a rudimentary DL (Anderson 1977; his figs. 28–30) (Table 2).

The reported range in lobe numbers of Goette's larvae may be attributed to methodological differences (distinction between LLs and the posterior body end is difficult and easily overlooked with light microscopy), species‐specific variation or differences in the examined developmental stage. We suggest subdividing Goette's larva into two distinct types. The first type is the classic four‐lobed type, which is characterised by an OH, a DL and two VLLs. The second type is the six‐lobed type, which is distinguished by an OH, a DL, two VLLs and two additional LLs. In this vein, we suggest that the so‐called two‐eyed Müller's larva of the acotylean *Hoploplana inquilina* (Surface 1907; Boyer et al. 1996, 1998) may rather be called a Goette's larva, as the number of eyes and the overall shape of the larva are rather suggestive of Goette's larva. Overall, this indicates a fluid distinction between Müller's and Goette's larvae, and it is not a big leap to imagine a Müller's larva as an eight‐lobed Goette's larva, as already suggested by Lang (1884). However, further research is required to fully understand the underlying mechanisms and evolutionary implications of lobe variation in different polyclad larvae.

### 4.3 Comparison of the Ultrastructure of the Apical Organ

Our TEM analysis of the apical organ of Goette's larvae in *S. pilidium* has supported the presence of three main cell types as key components of polyclad larval apical organs also described in other polyclad larval types: ATG cells, ATS cells and ATAn cells (Dittmann et al. 2023, 2024; Lacalli 1982, 1983; Ruppert 1978). However, variations occur in cellular arrangements across polyclad larval types.

#### 4.3.1 ATAn Cells

Based on the ultrastructural data of Ruppert (1978) (his fig. 4b), Dittmann et al. (2023) hypothesise the possibility of more than three anchor cells in Goette's larvae, though we observed only two in *S. pilidium*. This discrepancy could reflect species‐specific variation. The two ATAn cells observed anchor the apical organ in the epidermal layer, aligning with other studied polyclad larvae (Dittmann et al. 2023, 2024).

#### 4.3.2 ATG Cells

ATGs in Goette's larvae consist of ATG1, ATG2 and ATG3 cells. These cell types were found to extend to a different degree, with ATG1 and ATG2 spreading dorsally and ventrally and ATG3 remaining within the epidermal layer. No evidence of a DATC was found in Goette's larvae (Ruppert 1978 and this study). The DATC, first described in Müller's larvae of *P. crozieri* (Dittmann et al. 2023), is an additional ATC located at the anterior tip of the larvae, dorsal to the ATC. It consists of ciliated ampullary sensory neurons (SNs) and ATG cells, arranged in a semicircle dorsal to the ATC (Dittmann et al. 2023). A DATC‐like structure was also found in Curini‐Galletti's larvae of *T. mediterranea*, but it is located ventral to the ATC (Table 3 and Dittmann et al. 2024). As this study focused on the ultrastructure of 1–3 day‐old *S. pilidium* Goette's larvae, further research across various larval stages is needed to determine whether a second ATC may develop in later stages of Goette's larvae (Dittmann et al. 2023, 2024; Lacalli 1982; Ruppert 1978).

**Table 3:** Comparison of the apical organ between different polyclad larval types (adapted from Dittmann et al. 2024).

Species	Curini‐Galletti's larva	Goette's larva		Müllers' larva		
	*Theama mediterranea* 1	Undetermined^2^	*Stylochus pilidium* 3	Undetermined^2^	*Pseudoceros canadensis* 4	*Prostheceraeus crozieri* 5
ATAn	2	≥ 3	2	?	?	2
ATG1 cells	6	≥ 2	2	≥ 2	≥ 2	6
	Unfurcated	?	Furcated	?	Furcated	Furcated
	Lateral to brain	Ventral and dorsal	Dorsal	Ventral and dorsal	?	Lateral to brain
ATG1 microtubules	Peripheral	Without	Peripheral	Peripheral	?	Peripheral
ATG1 granules	436 ± 86 nm	591 ± 130 nm	423 ± 75 nm	816 ± 210 nm	774 ± 137 nm	450–650 nm
	Not electron‐dense	Electron‐dense	Not electron‐dense	Not electron‐dense	Not electron‐dense	Not electron‐dense
ATG2 cells	≥ 1	?	1	?	?	≥ 1
	Furcated	?	Furcated	?	?	Furcated
ATG2 microtubules	Without	?	Peripheral	?	?	Peripheral
ATG2 granules	136 ± 15 nm	?	434 ± 138 nm	?	?	150–350 nm
	Electron‐dense	?	Electron‐dense	?	?	Electron‐dense
ATG3/DATG cells	2	?	≥ 1	?	?	4
	Furcated	?	?	?	?	Furcated
ATG3/DATG microtubules	Without	?	Peripheral	?	?	Peripheral
ATG3/DATG granules	228 ± 38 nm	?	601 ± 117 nm	?	?	450–750 nm
	Electron‐dense	?	Electron‐dense	?	?	Electron‐dense
ATS cells	2	≥ 3	6	6	5	5
	Multiciliated	Multiciliated	Multiciliated	Monociliated	Monociliated	Monociliated
SN	Present	?	?	?	?	Present
	Without lumen	?	?	?	?	With lumen
	Ventral to ATC	?	?	?	?	Dorsal to ATC

^1^ Dittmann et al. 2024.

^2^ Ruppert 1978.

^3^ This study.

^4^ Lacalli 1982, 1983.

^5^ Dittmann et al. 2023.

Curini‐Galletti's, Goette's and Müller's larvae share a similar overall organisation of their apical organ—the centre made of ATS cells, surrounded by ATG1 cells which are embedded in ATAn cells and two other ATG cells are present—but still some variations regarding the number of cells and their arrangement exist (Table 3).

Ruppert (1978) identified cells around the ATC (his fig. 4b) as rhabdites. We hypothesise that these cells are likely ATG3 cells and not rhabdites. Rhabdites have electron‐dense granules with concentric layers. In contrast, ATG3 granules are smaller, less dense, lack concentric structures and contain peripheral microtubules (Martin 1978; Smith et al. 1982).

#### 4.3.3 ATS Cells

A key difference between the larval types is the number and ciliation of ATS cells. In Curini‐Galletti's larvae, two multiciliated ATS cells were identified. Goette's larvae also show multiciliation but have six ATS cells. In contrast, Müller's larvae have monociliated ATS cells with 5–6 nuclei, depending on the species (Dittmann et al. 2023, 2024; Lacalli 1982; Ruppert 1978) (Table 3).

### 4.4 Homology or Convergence Between Different Polyclad Larval Types

**Figure 8:**
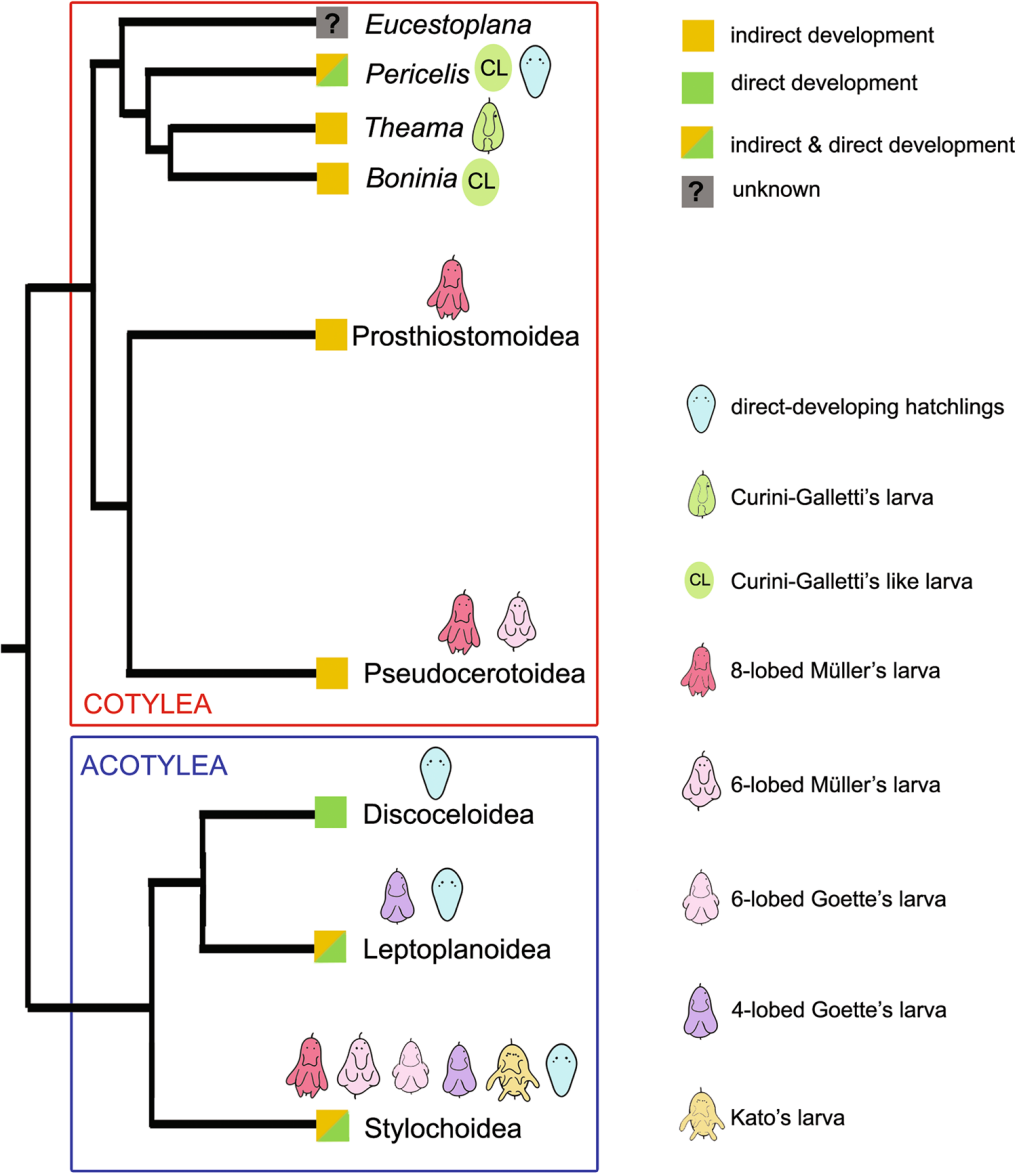
Cladogram of the interrelationships of major polyclad taxa modified after Goodheart et al. (2023) and Dittmann et al. (2024), showing the reported larval types for the respective groups. Pictograms of larvae were modified from Lang (1884) and Kato (1940). Adapted with permission from Gotsis (2024).

Polycladida are distinctive among free‐living flatworms due to their dual development modes: direct and indirect. This raises the question of which mode represents the ancestral state (Goodheart et al. 2023). Goodheart et al. (2023) present two main evolutionary scenarios for the origin of indirect development (Figure 8). In the first, Müller's larva is proposed as the ancestral polyclad larval form, with Goette's larva evolving from it. The second hypothesis reverses this, suggesting Goette's larva as the ancestral form, with Müller's larva emerging independently multiple times. Additionally, a third scenario suggests that Curini‐Galletti's larva may be the ancestral form (Dittmann et al. 2024), although a smooth transition between ‘distinct’ larval types can be considered (see Section 4.2). Another possibility is that polyclad larvae are not ancestral at all, but instead evolved independently multiple times (Goodheart et al. 2023).

The ultrastructural and compositional similarities between the apical organs of the studied three polyclad larval types highly suggest a common origin of these larva: they all feature a strongly glandular apical organ with three gland cell types, ciliated ATS cells and ATAn cells (Figure 7), also referred to as the ATC (Dittmann et al. 2023, 2024). While in Curini‐Galletti's larva and Müller's larva, another ATC completes the apical organ (Dittmann et al. 2023, 2024), a missing additional ATC in Goette's larva shows that it is not a universal feature among polyclad larvae. More comparative data from other polyclad larvae, and also from direct developing juveniles, may clarify whether a second ATC was possibly lost in Goette's larva.

### 4.5 Homology or Convergence Between Polyclad and Other Spiralian Larvae

The idea that polyclad larvae are derived from spiralian larvae implies at least four independent losses of a planktonic larval development within Platyhelminthes (in Catenulida, Macrostomorpha, Prorhynchida and Euneoophora) (Laumer et al. 2015). However, there is increasing evidence for the homology of polyclad larvae with trochophore larvae due to similarities in ciliary bands, spiral cleavage patterns and cell fates (Nielsen 2018; Wu et al. 2020). To address the question of homology or convergence, comparing larval features is key. The apical organ is a reliable feature for such analyses due to its complexity and consistency across polyclad species (Dittmann et al. 2023; Wanninger 2008). The presence of an apical organ in various trochophore larvae is well documented (Bleidorn 2019; Dittmann et al. 2023; Marlétaz et al. 2019; Nielsen 2018). The new ultrastructural data presented here show that the apical organ of the acotylean Goette's larva shares similarities with the cotylean apical organ, indicating homology within polyclad larval types. Dittmann et al. (2023, 2024) identified conserved cellular components in polyclad larvae that are homologous to those observed in other spiralian taxa. These findings could challenge the prevailing view that larval development is an apomorphy of Polycladida, instead supporting the idea of larval development as the ancestral state not only for Platyhelminthes, but possibly across the spiralian lineage.

## Author Contributions

**Davina Düngler:** conceptualisation, methodology, investigation, imaging, visualisation, validation, writing. **Clemens Gotsis:** investigation, validation, imaging, visualisation, writing. **Isabel L. Dittmann:** conceptualisation, supervision, resources, methodology, investigation, imaging, validation, writing. **Stefan Redl:** technical support, methodology, imaging, resources, writing. **Michael W. Hess:** resources. **Bernhard Egger:** conceptualisation, sampling, supervision, resources, investigation, validation, writing.

## Ethics Statement

Flatworms are not regulated in any European Parliament directives, but we have taken special care to avoid the potential suffering of animals.

## Conflicts of Interest

The authors declare no conflicts of interest.

## Data Availability

The data that support the findings of this study are available from the corresponding author upon reasonable request.
